# Reconfiguration of N Metabolism upon Hypoxia Stress and Recovery: Roles of Alanine Aminotransferase (AlaAT) and Glutamate Dehydrogenase (GDH)

**DOI:** 10.3390/plants5020025

**Published:** 2016-05-31

**Authors:** Houssein Diab, Anis M. Limami

**Affiliations:** University of Angers, UMR 1345 IRHS, SFR 4207 QUASAV, 2 Bd Lavoisier, F-49045 Angers, France; drhd85@hotmail.com

**Keywords:** alanine, alanine aminotransferase (AlaAT), glutamate, glutamate dehydrogenase (GDH), glycolysis, hypoxia, nitrogen

## Abstract

In the context of climatic change, more heavy precipitation and more frequent flooding and waterlogging events threaten the productivity of arable farmland. Furthermore, crops were not selected to cope with flooding- and waterlogging-induced oxygen limitation. In general, low oxygen stress, unlike other abiotic stresses (e.g., cold, high temperature, drought and saline stress), received little interest from the scientific community and less financial support from stakeholders. Accordingly, breeding programs should be developed and agronomical practices should be adapted in order to save plants’ growth and yield—even under conditions of low oxygen availability (e.g., submergence and waterlogging). The prerequisite to the success of such breeding programs and changes in agronomical practices is a good knowledge of how plants adapt to low oxygen stress at the cellular and the whole plant level. In the present paper, we summarized the recent knowledge on metabolic adjustment in general under low oxygen stress and highlighted thereafter the major changes pertaining to the reconfiguration of amino acids syntheses. We propose a model showing (i) how pyruvate derived from active glycolysis upon hypoxia is competitively used by the alanine aminotransferase/glutamate synthase cycle, leading to alanine accumulation and NAD^+^ regeneration. Carbon is then saved in a nitrogen store instead of being lost through ethanol fermentative pathway. (ii) During the post-hypoxia recovery period, the alanine aminotransferase/glutamate dehydrogenase cycle mobilizes this carbon from alanine store. Pyruvate produced by the reverse reaction of alanine aminotransferase is funneled to the TCA cycle, while deaminating glutamate dehydrogenase regenerates, reducing equivalent (NADH) and 2-oxoglutarate to maintain the cycle function.

## 1. Introduction 

The absence of a tissue or a system dedicated to oxygen uptake and delivery to the organs in plants may create localized hypoxic environments when oxygen diffusion is hindered by the anatomy and the structure of the tissue—e.g., dense cell packing. Developing and germinating seeds, tubers, bulky fruits, meristems, germinating pollen, and the phloem are the organs the most likely to lack oxygen even when the whole plant is growing in aerobic conditions [1]. Environmental conditions like flooding or soil waterlogging are the other major causes of more or less prolonged periods of hypoxia/anoxia due to the slow diffusion of oxygen in water and competition of the roots with respiring microorganisms. In the context of climatic change, arable farmland productivity is threatened by more frequent heavy precipitation causing submergence and waterlogging of plants; furthermore, the fact that cultivated crops were not selected to cope with oxygen limitation is an aggravating condition [2,3,4]. Consequently, like the adaptation of plants to drought and saline stress, low oxygen stress deserves more interest from the scientific community. Low oxygen sensing, signaling, and adaptation of plants to its limitation at both the cellular and whole plant levels should be investigated more thoroughly, and the derived knowledge should be taken into account in breeding programs and agronomical practices for saving plant fitness, growth, and development even when oxygen availability is low [4]. In the present article, we will first give an overview of the recent knowledge on low oxygen sensing and signaling in plants and adaptation by metabolic adjustment to submergence- or waterlogging-induced hypoxia/anoxia. Secondly, we will focus on the major changes pertaining to the reconfiguration of amino acids metabolism, highlighting the role of alanine that accumulates during periods of stress as a C and N storage compound readily utilizable during the post-hypoxia recovery period.

## 2. The Role of Metabolic Adjustment in Cellular Response to Low Oxygen Availability

Hypoxia-induced inhibition of mitochondrial oxidative phosphorylation results in an energy crisis due to the insufficient ATP production to face the energy requirement of cellular processes [5]. Microarray-based transcriptome analyses of 21 organisms representing the four kingdoms of plants, animals (including *Homo sapiens*), fungi, and bacteria submitted to varying degrees of oxygen deficiency showed that metabolic changes in relation to energy production (fermentation) and utilization are among the conserved responses across the four kingdoms [6]. The nature of these changes suggests that they are aimed at counteracting the damaging effects of energy crisis. In *Arabidopsis*, translatome analysis revealed a group of 49 genes—actually named the core-hypoxia-responsive genes—which are prioritized for translation in response to low oxygen stress. Several enzymes associated with the reconfiguration of carbon metabolism are members of this group—e.g., pyruvate decarboxylase (PDC1 and PDC2), alcohol dehydrogenase (ADH1), and sucrose synthase (SUS4) [7]. Analysis of the transcriptome of rice coleoptile, in a variety adapted to elongate under anoxia, showed (similarly to *Arabidopsis*) the induction of the above mentioned genes, and very interestingly it showed that genes encoding enzymes of pyruvate and phosphoenolpyruvate (PEP) metabolism are those that are the most affected [8]. The transcription of genes encoding pyruvate phosphate dikinase (PPDK) and PEP carboxykinase (PCK) were strongly increased, and the gene encoding PEP carboxylase (PEPC) was strongly inhibited, while the expressions of genes encoding pyruvate kinase (PK) and pyruvate dehydrogenase (PDH) were hardly changed [8]. In total, activation of the alcoholic fermentation pathway allows for NAD^+^ regeneration to maintain glycolysis and ATP production [9,10,11,12], and for saving ATP, PPi-dependent enzymes are preferred to ATP-dependent enzymes. This strategy is illustrated by the fact that SUS is preferred to invertase, PPi-dependent phosphofructokinase (PFP) is preferred to phosphofructokinase (PFK) for F-1-6-bis-P synthesis and PPDK is preferred to pyruvate kinase (PK) for pyruvate synthesis [13,14].

Induction and coordination of the expression of low oxygen stress-responsive genes—including those involved in primary metabolism and energy homeostasis under hypoxic and anaerobic conditions—were shown to be under the control of transcription factors belonging to the group-VII Ethylene Response Factors (ERFs) RAP2.2, RAP2.3, RAP2.12, HRE1, and HRE2; for review, see [1,2,15]. Recently, RAP2.2 and RAP2.12 were shown to act redundantly as principal activators of low oxygen stress-inducible genes and RAP2.3 contributes to this redundancy [16]. The group VII ERFs involved in low oxygen stress are subjected to oxygen-dependent posttranslational modification through the N-end rule pathway (NERP) for protein destabilization [17,18,19]. It is proposed that as cellular oxygen concentration decreases, RAP2.2 and RAP2.12, which are constitutively expressed, would escape post-translational modification and proteolysis via the NERP before being transported to the nucleus where they stimulate the expression of secondary ERFs such as *HRE1* and *HRE2*, allowing then for several down-stream hypoxia-response genes to be expressed [20,21]. Upon the return to normoxia, all the members of the group VII ERFs are subjected to the NERP-mediated degradation. *Arabidopsis* double mutant affected in both Arginyl-tRNA transferase 1 and 2 (*ate1/ate2*) and the mutant affected in N-recognin PROTEOLYSIS 6 (*prt6*) unable to run NERP-mediated protein degradation exhibited constitutive expression of marker genes of hypoxic and anaerobic metabolism (*ADH*, PDC, *SUS*) as well as more than half of the 49 core hypoxia-induced genes [17]. It has been shown that if overexpression of the native *RAP2.12* improves survival under low oxygen stress, the constitutive accumulation of versions of RAP2.12 insensitive to NERP proteolysis decreased survival, indicating that a fine-tuning of transcription is a prerequisite for cellular homeostasis under hypoxia. Interestingly, it has been recently demonstrated in *Arabidopsis* that RAP2.12 is negatively regulated through a protein–protein interaction with a hypoxia-inducible transcription factor that encodes a trihelix DNA-binding protein named Hypoxia Response Attenuator (HRA1). In addition to its negative regulation of RAP2.12, HRA1 negatively regulates activation of its own promoter. Finally, it is worth noting that HRA1 is transcriptionally activated by RAP2.12 upon stabilization. It appears then that HRA1 and RAP2.12 constitute a control unit that allows plants to modulate the extent of the response to hypoxia, including anaerobic enzyme production to levels that improve low oxygen stress endurance [22].

The cross kingdom comparison of transcriptomic adjustment to low oxygen stress highlighted plant-specific responses; in plants, ethanol rather than lactate fermentation regenerates NAD^+^. This regeneration is crucial to maintain higher rates of glycolysis. Efficiency of this strategy for ATP production is very low, it is carbohydrate-costly and it drains the carbon store of the cell very quickly, leading to carbon starvation and cellular death. Therefore, it appears that management of carbon reserves is crucial for survival upon hypoxia [9]. This explains why the ability to supply the roots with carbohydrates—the substrate of alcohol fermentation during prolonged periods of soil hypoxia/anoxia—appeared as a determinant in waterlogging tolerance, as well illustrated in the case of common ash (*Fraxinus excelsior*) [23]. Similarly, hypoxia/anoxia-sensitive seeds of wheat and barley do not induce amylases under hypoxic/anoxic conditions of germination, in contrast to anaerobic germination-competent rice seeds [24]. Another specificity of the plants is the reconfiguration of nitrogen metabolism, in particular amino acids metabolism via alanine fermentative pathway [15,25,26].

## 3. Alanine Aminotransferase Safeguard Carbon in a Nitrogen Store upon Hypoxia

Alanine fermentative pathway does not regenerate NAD^+^, and unlike several metabolites known for either their signaling or protective role upon abiotic stresses—e.g., proline, betaine; alanine intrinsically may not play a protective role in hypoxic stress [3,10,27]. Nevertheless, alanine was shown to accumulate in various plant species under hypoxia/anoxia conditions. Furthermore, in *Arabidopsis* plantlets submitted for 2 h to anoxic atmosphere (99.99% argon) *AlaAT* (alanine aminotransferase; At1g17290) was the only nitrogen metabolism gene among the core hypoxia-responsive genes [7]. *AlaAT* expression was induced in *Arabidopsis* under similar or even less-drastic oxygen stress—e.g., in hairy roots soaked in hypoxic (0.5% O_2_) liquid medium [28], in young seedlings soaked in hypoxic (3% O_2_) [13] or anoxic (90% N_2_ 10% H_2_) [29] liquid medium, and in plantlets grown on solid medium under anoxic (99.99% argon), or hypoxic (1% or 1.5% O_2_) atmosphere [30,31]. In legume species *Medicago truncatula*, *Lotus japonicas*, and *Glycine max*, either AlaAT activity, gene expression, or both were shown to increase in the roots of waterlogged plants [25,26,27,32]. Insight on the regulation of the expression of *AlaAT* under low oxygen stress was gained by browsing public transcriptomic data of *Arabidopsis* mutants affected in the expression of genes encoding members of the group VII ERFs transcription factors and genes encoding enzymes necessary for NERP-mediated degradation of proteins [15]. From this investigation, it appeared that, similarly to hypoxia marker genes *ADH1* (At1g77120) and *PDC1* (At4g33070), the expression of *AlaAT* (At1g17290) was affected in mutants either overexpressing *RAP2.2* or Knocked out in its expression [30]. Compared to the wild-type, the expression of *AlaAT* was strongly increased in the shoots of the *RAP2.2* overexpressors, but only under hypoxia in the dark and not under hypoxia in the light or normoxia in the dark [30]. Unlike the above-mentioned hypoxia marker genes, both *AlaAT* genes (At1g17290 and At1g72330) were not up-regulated under non-stress conditions in the mutants defective in NERP-mediated degradation of proteins (*ate1/ate2* and *prt6*) under normoxia or hypoxia (2 h) [17]. Finally, unlike several hypoxia-regulated genes, *AlaAT* was not over-induced by flooding in *Arabidopsis* mutants overexpressing *RAP2.12* [33]. Altogether, these data show that *RAP2.2* is necessary but not sufficient for hypoxia-induced *AlaAT* in the shoots of *Arabidopsis*. A yet-unknown dark stress-dependent element seems to be necessary in conjunction with RAP2.2 for the induction of *AlaAT* by hypoxia (see hypothetical cross-talk between hypoxia- and darkness-induced signaling pathways for transcriptional regulation of *AlaAT* in [33]). These data also suggest that *AlaAT* regulation might not be dependent on NERP for oxygen sensing in plants.

**Figure 1:**
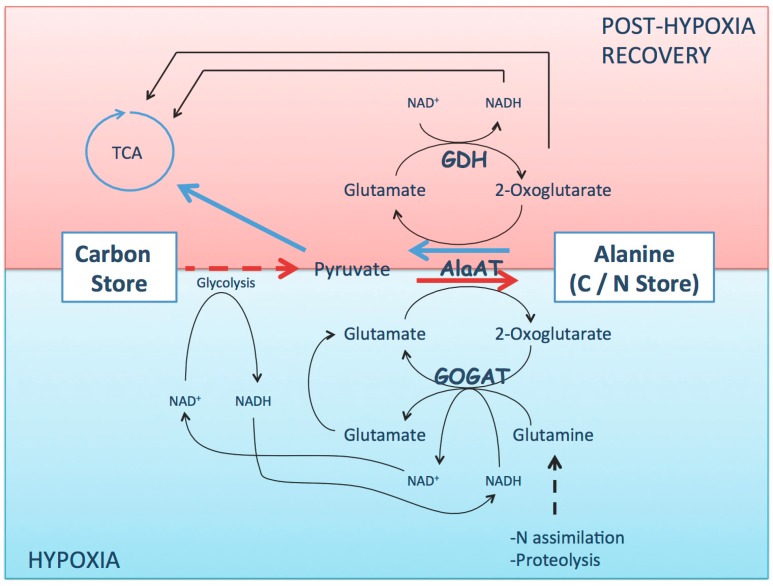
Schematic representation of the central role of alanine during hypoxia and post-hypoxia recovery periods. **HYPOXIA:** Visualization of carbon flux from carbon storage compounds to alanine through AlaAT/NADH-GOGAT cycle. (POST-HYPOXIA) Visualization of alanine mobilization through AlaAT/GDH cycle and carbon flux towards TCA cycle. Scheme drawn by integrating data from transcriptomic and metabolomic studies and studies combining ^15^N and ^13^C labeling in various species such as *Medicago truncatula*, *Lotus japonicus*, *Glycine max* and *Arabidopsis* *thaliana*. AlaAT: Alanine aminotransferase; GDH: Glutamate Dehydrogenase GOGAT: Glutamne Oxoglutarate Aminotransferase.

Reconfiguration of primary C/N metabolism upon hypoxia was investigated *in vivo* in *Medicago truncatula*, *Lotus japonicas*, and *Glycine max* through experiments using ^15^N and ^13^C isotope labeling and metabolomics approach [26,34,35]. Altogether, these studies allow the conclusion that the reversible reaction of the interconversion of pyruvate and glutamate to alanine and 2-oxoglutarate is the main route of alanine synthesis under hypoxia (Figure 1). GABA transaminase (GABA-T) that can use either pyruvate or 2-oxoglutarate as amino acceptor seems to preferentially use pyruvate under hypoxic conditions, which constitutes an alternative pathway for alanine accumulation. However, *Arabidopsis* GABA-T null mutants accumulated only slightly less alanine than wild-type upon hypoxia [36,37]. AlaAT activity was investigated *in vivo* by feeding *Medicago truncatula* seedlings with either ^15^N-glutamate or ^15^N-alanine; upon hypoxia, AlaAT activity was directed towards alanine synthesis using glutamate as amino donor, while the reverse reaction of glutamate synthesis using alanine as amino donor was inhibited [32]. Labeling *Medicago truncatula* [32] and *Glycine max* [25] with ^15^NH_4_^+^ showed that after 24 h, alanine accumulated as the major labelled amino acid in hypoxic tissues. Interestingly, ^15^N-glutamine, ^15^N-asparagine, and ^15^N-aspartate were dramatically low, while ^15^N-glutamate was synthesized at levels close to that in the control with the pool of ^15^N-glutamate remaining stable. ^15^NH_4_ and ^13^C-Glutamate labeling data in *Glycine max* showed that under hypoxia, the label accumulated in alanine, glutamate, and 2-oxoglutarate at higher concentrations than that in the control, while the opposite was true for glutamine. In the same study, ^15^N-GABA was significantly higher than in the control, and ^13^C-Glutamate labelling revealed considerable GABA shunt activity that explained the increase in the redistribution of ^13^C to succinate [25]. Altogether, these results suggest that rather than just an activation of alanine synthesis, amino acids metabolism is deeply affected by oxygen limitation and energy shortage. Limami *et al.* [34] suggested that the major reconfiguration of amino acids metabolism under hypoxia consisted of a concerted modulation of nitrogen flux through the pathways of both alanine and glutamate synthesis. The ATP-consuming enzymes glutamine synthetase (GS) and asparagine synthetase (AS) were significantly inhibited—probably as part of a cellular strategy to mitigate the damaging effect of energy crisis. Regeneration of glutamate as a substrate of AlaAT and glutamate decarboxylase (GDC) is likely to occur through the reductive amination of 2-oxoglutarate by Glutamine Oxoglutarate amino transferase (GOGAT). Through a ^13^C-Pyruvate feeding experiment in soybean, it was proposed that NADH-GOGAT uses glutamine along with ^13^C-Oxoglutarate to synthesize a mixture of ^13^C-Glutamate and ^12^C-Glutamate [25]. In *Medicago truncatula*, the inhibition of GOGAT with azaserine disturbed alanine accumulation and blocked germination and seedling establishment under hypoxia/anoxia [27].

In total, it appears that changes in amino acids metabolism may contribute in several manners to mitigate damaging consequences of oxygen limitation. Upon hypoxic stress, in roots, NADH-GOGAT activity contributes to the regeneration of NAD^+^ that helps glycolysis to proceed [34]. Through alanine shunt, synthesis of alanine generates 2-oxoglutarate, which can be further metabolized to succinate via the TCA cycle enzyme succinate CoA ligase, thus providing additional ATP per molecule of sucrose metabolized [26]. Alanine synthesis saves carbon by competing with ethanol synthesis for the common precursor pyruvate. Ethanol is a dead-end product that leaks out of the tissue, representing a net loss of carbon [9,11]. Storing nitrogen in the form of alanine through the NADH-GOGAT/AlaAT cycle saves ATP that is otherwise necessary for assimilating mineral nitrogen as glutamine and asparagine via ATP consuming enzymes (Figure 1).

## 4. Alanine Aminotransferase/Glutamate Dehydrogenase Cycle Mobilizes Carbon from the Nitrogen Store upon Reoxygenation

In plants, GDH was shown to operate mainly in the direction of glutamate deamination to provide carbon skeletons and reducing equivalents [38,39,40,41]. In *Arabidopsis*, GDH gene expression and enzyme deaminating activity were boosted by the exposure of plants to several days of darkness that lead to severe carbon shortage [38,42,43,44,45]. In *gdh1-2-3* *Arabidopsis* triple mutants deprived of GDH activity, alanine and GABA accumulated in the roots where GABA shunt was activated to compensate for the lack of NADH-GDH as a provider of carbon to the TCA cycle [38].

Involvement of GDH in low oxygen stress received little attention compared to that in other abiotic stresses. Still, browsing public transcriptomic data of *Arabidopsis* submitted to various conditions of low oxygen stress showed that the expression of glutamate dehydrogenase isogene *GDH1* (At5g18170) and *GDH2* (At5g07440) was affected; *GDH2* expression increased after 30 min of exposure to hypoxia (0.5% O_2_) [28], *GDH1* and *GDH2* were up-regulated by 8 h of anoxia (atmosphere of 100% N_2_) [46] and *GDH1* and *GDH2* [29] were up-regulated by 6 h of anoxia (90% N_2_, 10% H_2_) in darkness. All these experiments were run on media supplemented with sucrose, a condition that rules out an induction of GDH genes expression only as a consequence of hypoxia/anoxia-induced carbon shortage. In *Medicago truncatula* submitted to waterlogging-induced hypoxia, the expression of *GDH1* (Medtr7g085630) was strongly up-regulated similarly to that of the mitochondrial *AlaAT2* (Medtr8g023140) [34]. The question of whether GDH would exceptionally, under low oxygen stress, operate in the direction of glutamate synthesis (and by doing so participate to the regeneration of NAD^+^) was addressed in *Medicago truncatula*. Feeding the seedlings ^15^NH_4_^+^ in the presence and absence of the GS-inhibitor methionine sulfoximine (MSX) under hypoxic condition showed clearly that GDH activity was very low compared to normoxic condition and was not contributing to glutamate synthesis [34]. The discrepancy between GDH gene expression and enzyme activity suggests that the induction of the gene encoding GDH upon hypoxia may be interpreted as anticipation of the return to aerobic conditions. It has been observed that the return to aerobic conditions was anticipated in plants subjected to hypoxic stress by expressing genes whose products have functions during the subsequent recovery period [10]. In this case, GDH’s main role would be the regeneration of 2-oxoglutarate by deaminating glutamate during the post-stress recovery period (Figure 1) [34]. This role of GDH during reoxygenation is further supported by the observation in *Arabidopsis* that *GDH1* and *GDH2* were induced to much higher levels during reoxygenation than during anoxia. In addition, a third isogene *GDH3* that was not affected by low oxygen stress was up-regulated specifically during reoxygenation [46]. Interestingly, in this study, *AtAlaAT1* and *AtAlaAT2* were also re-induced during reoxyganation [46]. The hypoxia-inducible isognene *AlaAT1* in *Arabidopsis* showed a similar pattern of regulation and activity [36]. Expression of the gene encoding AlaAT1 was up-regulated by hypoxic stress, while the activity of the enzyme was shown to be the conversion of alanine into pyruvate and glutamate during the post-hypoxic period. Finally, a non-plant model—that is, GDH from the tail muscle of the freshwater crayfish *Orconectes virilis*—supports the idea of the involvement of GDH during the return to normoxia. In this animal it was shown that GDH is submitted to a posttranslational regulation—phosphorylation of the enzyme inhibits its activity under oxygen limitation until the return to normoxia [47].

## 5. Conclusions

Altogether, these results allow the suggestion of a model of storage and mobilization of carbon during the transition from hypoxia to post-hypoxia stress (Figure 1). During hypoxia stress, pyruvate as a byproduct of glycolysis is competitively used through an AlaAT/NADH-GOGAT cycle, leading to the storage of carbon in alanine. Alanine is synthesized by AlaAT using glutamate as an amino donor, while NADH-GOGAT uses 2-oxoglutarate to regenerate glutamate and NAD^+^. During the post-hypoxia recovery period, carbon is mobilized through an AlaAT/GDH cycle. Alanine is deaminated by the reverse reaction of AlaAT to produce pyruvate and glutamate. Carbon is funneled to the TCA cycle as pyruvate, while GDH, by deaminating glutamate, generates 2-oxoglutarate to maintain the cycle function and produce reducing equivalent (NADH).
